# Diabetes and hypertension guidelines and the primary health care practitioner in Barbados: knowledge, attitudes, practices and barriers-a focus group study

**DOI:** 10.1186/1471-2296-11-96

**Published:** 2010-12-03

**Authors:** O Peter Adams, Anne O Carter

**Affiliations:** 1 Faculty of Medical Sciences, The University of the West Indies, Cave Hill Campus, St. Michael, Barbados; 2 Department of Community Health and Epidemiology, Queen's University, Ontario, Canada

## Abstract

**Background:**

Audits have shown numerous deficiencies in the quality of hypertension and diabetes primary care in Barbados, despite distribution of regional guidelines. This study aimed to evaluate the knowledge, attitudes and practices, and the barriers faced by primary care practitioners in Barbados concerning the recommendations of available diabetes and hypertension guidelines.

**Methods:**

Focus groups using a moderator's manual were conducted at all 8 public sector polyclinics, and 5 sessions were held for private practitioners.

**Results:**

Polyclinic sessions were attended by 63 persons (17 physicians, 34 nurses, 3 dieticians, 3 podiatrists, 5 pharmacists, and 1 other), and private sector sessions by 20 persons (12 physicians, 1 nurse, 3 dieticians, 2 podiatrists and 2 pharmacists).

Practitioners generally thought they gave a good quality of care. Commonwealth Caribbean Medical Research Council 1995 diabetes and 1998 hypertension guidelines, and the Ministry of Health 2001 diabetes protocol had been seen by 38%, 32% and 78% respectively of polyclinic practitioners, 67%, 83%, and 33% of private physicians, and 25%, 0% and 38% of non-physician private practitioners. Current guidelines were considered by some to be outdated, unavailable, difficult to remember and lacking in advice to tackle barriers. Practitioners thought that guidelines should be circulated widely, promoted with repeated educational sessions, and kept short. Patient oriented versions of the guidelines were welcomed.

Patient factors causing barriers to ideal outcome included denial and fear of stigma; financial resources to access an appropriate diet, exercise and monitoring equipment; confusion over medication regimens, not valuing free medication, belief in alternative medicines, and being unable to change habits. System barriers included lack of access to blood investigations, clinic equipment and medication; the lack of human resources in polyclinics; and an uncoordinated team approach. Patients faced cultural barriers with regards to meals, exercise, appropriate body size, footwear, medication taking, and taking responsibility for one's health; and difficulty getting time off work to attend clinic.

**Conclusions:**

Guidelines need to be promoted repeatedly, and implemented with strategies to overcome barriers. Their development and implementation must be guided by input from all providers on the primary health care team.

## Background

Diabetes mellitus and hypertension cause a significant burden of disease in Barbados [1-3]. Audits of primary care have shown numerous deficiencies in the quality of hypertension and diabetes care [4-6]. In an attempt to produce a higher quality of care the Commonwealth Caribbean Medical Research Council now called the Caribbean Health Research Council (CCMRC and CHRC respectively) developed and distributed practice guidelines. *Managing Diabetes in Primary Care *was produced in 1995 [7] and *Managing Hypertension in Primary Care in the Caribbean *in 1998 [8]. Subsequent audits [4,9,10] showed only limited improvement. Updated versions of the CHRC guidelines were released in 2006 and 2007 [11,12]. Strong implementation strategies did not accompany release of any of the CCMRC/CHRC guidelines. They consisted of a pair of workshops attended by some health care practitioners. In addition in 2001 the Ministry of Health of Barbados developed the *Protocol for the Monitoring, Surveillance and Management of Diabetes Mellitus in Barbados *[13], which was implemented by seminars directed at public sector health professionals.

When acted upon, guidelines have been shown to have potential to improve both the process of care and patient health outcomes [14,15]. However, the actual value of guidelines has seldom been assessed through formal evaluation procedures [16-18], and when they are evaluated, they are often found to fall short of expectations [14,17,19]. A great deal rests on the quality of the implementation strategies. Didactic lecture-based CME and mailed unsolicited materials are weak methods, while audit and feedback delivered by peers or opinion leaders, reminder systems and academic detailing are strong methods. Multiple simultaneous interventions are the strongest implementation method [20]. An assessment of the needs and barriers faced by practitioners and patients is valuable in assisting in the design of an effective implementation strategy.

Before a guideline can affect patient outcomes it must first affect practitioner knowledge, then attitudes and finally behaviour. Practitioners need to first become aware of the existence of the guideline, and then familiar with its recommendations. Attitudes required include agreement with the recommendations, self-efficacy (the belief that one can perform the required behaviour), outcome expectancy (the expectation that a given behaviour will produce a particular outcome) and motivation to change current practice [21]. Even with the correct attitude, barriers which could be guideline, patient, practitioner, system or society related may prevent guideline adoption.

The objectives of this study were to evaluate the knowledge, attitudes and practices, and the barriers faced by primary care practitioners in Barbados concerning the recommendations of available diabetes and hypertension guidelines and protocols by means of focus groups.

## Methods

### Setting

Eight publicly funded polyclinics strategically located around the island provide free comprehensive primary care, while in 2005 at least 89 private general practitioners provided service for a fee. At public sector polyclinics patients are often seen by a nurse before the consultation with the general practitioner. A dietician and podiatrist are available at each clinic on specific days. All polyclinics have a pharmacy. Most private practitioners work in solo or small group practices, and do not employ a nurse. Robust data is not available but it has been estimated that primary care is approximately equally split between the public and private sectors [22]. Patients in both sectors are provided, at no cost under the Barbados Drug benefit Service, with an appropriate range of medications to treat diabetes, and hypertension. Otherwise private patients pay for all services.

### Focus group recruiting-public practitioners

Focus group sessions were held onsite at all 8 polyclinics during the afternoon when the workload was more likely to be light. All physicians and other practitioners providing diabetes and hypertension care, who were present in the polyclinic at the time of the session and were not occupied with essential tasks were invited to attend. Including non-physician practitioners reflected the team approach to diabetes care found in Polyclinics.

### Focus group recruiting-private practitioners

Private sector primary care physicians were selected from a previously validated list containing 89 names, and private sector dieticians, podiatrists/chiropodists, pharmacists and nurses identified from the yellow pages of the telephone book were contacted by telephone. The focus group process and goals of the study were described, and then the health care worker was invited to participate in a focus group to be held in the evening at a hotel or at the main hospital. Persons agreeing to participate were reminded on the day of the meeting.

### Focus group process

Following standard focus group methodology [23], a moderator's manual was prepared to meet the objectives of the study. It focussed on the practitioners' knowledge of the CCMRC diabetes and hypertension guidelines [7,8] and the Ministry of Health diabetes Protocol [13]; practices while caring for patients with diabetes and hypertension; attitudes to guidelines, the diseases diabetes and hypertension and to patients with these diseases; the barriers faced when trying to follow guidelines and in treating patients with these diseases; and recommendations for changes within and outside the health care sector that would help both practitioners and their patients to achieve better care and better health. Props for the focus groups included copies of the 1995 and 1998 CCMRC guidelines, the 2001 Ministry of Health diabetes Protocol, and drafts of the new CHRC guidelines which at the time were being developed (released subsequent to this study in 2006 and 2007).

The moderator's manual was pilot tested on a convenience sample of private and public sector practitioners and adjustments were made as necessary. Once finalized, the manuals were followed closely in each focus group but flexibility was allowed if new concepts or problems arose during a focus group session.

Focus group sessions were conducted in 2005. Two investigators, a facilitator (AOC) and a recorder (OPA), attended each session. On arrival participants were presented with a written sheet describing the focus group process, the goals and objectives of the study, and explaining that sessions would be taped but participants would remain anonymous. Participants' questions were answered, they were asked to sign a consent form, and then to complete an anonymous short questionnaire concerning their demographic details. The facilitator then started the session by reiterating the goals and the focus group process and explaining again the use of the tape recorder. Participants were again given an opportunity to ask questions. The facilitator then started the session questions, following the manual. The recorder took notes of the discussion. When the moderator's manual questions were completed, participants were thanked for their contribution and asked if they had any additional comments that they wished to make. The session then ended. Following each focus group session, a debriefing session was held to summarize findings, identify any problems and develop plans for future sessions. No revisions or additions needed to be made in the moderator's manual after sessions had started.

### Data analysis

Transcriptions of the tapes were made, and then the text was divided into sections dealing with each topic of interest. If there was difficulty understanding the tapes, the notes taken at the session were consulted. Each comment was given content codes to designate the content issues contained in the comment. Information from focus groups of private physicians and polyclinic practitioners were analysed separately and compared, and a summary was then done.

### Ethical approval

Approval was obtained from the Institutional Review Board of the University of the West Indies, Cave Hill Campus and the Ministry of Health, Barbados.

## Results

Thirteen focus group sessions were held. Each lasted approximately 2 hours and was attended by 2 to 10 practitioners. No attendee refused to participate.

Attending the 8 polyclinic sessions were 63 persons: 17 physicians (29% male, mean age 36), 34 nurses (all female, mean age 49), 3 dieticians (all female, mean age 34), 3 podiatrists (all female, mean age 39), 5 pharmacists (all female, mean age 34), and one female dental assistant. Of these, 10 (3 physicians) reported also having a private practice. The mix of polyclinic practitioners attending the focus groups was representative of polyclinic providers with a preponderance of nurses. Professional employee lists indicate that approximately 49% of the public sector doctors and 25% of the nurses were sampled [24].

Attending the 5 private provider sessions were 20 persons: 12 physicians (7 males, 5 females, mean age 45), 1 female nurse, 3 dieticians (all female, mean age 46), 2 podiatrists (all female, mean age 35), 2 pharmacists (all male, mean age 37). Four of the physicians in this group also worked in the polyclinics; the remaining participants were strictly in private practice. Physicians were representative of private practitioners (58% male, compared to 68% male on the list of physicians) with a somewhat older age and a higher proportion of males than in the polyclinics.

### Knowledge

CCMRC 1995 diabetes and 1998 hypertension guidelines, and the Ministry of Health 2001 diabetes protocol had been seen by 38%, 32% and 78% respectively of polyclinic practitioners, 67%, 83%, and 33% of private physicians, and 25%, 0% and 38% of non-physician private practitioners.

### Attitudes and Practices

Most private physicians had read the CCMRC guidelines but did not follow them because they were outdated, not patient centred, difficult to remember, and did not give advice on how to tackle barriers. "I may follow guidelines but not necessarily give good care because the focus must be on the patient not the diabetic." Polyclinic practitioners also thought that the guidelines were not sufficiently patient centred. However many who had read the guidelines found them helpful. Private physicians were more likely than polyclinic physicians to say they followed the WHO, American Diabetes Association and JNC guidelines. They preferred them because they were available online, updated regularly and promoted at conferences. The diabetes protocol was felt to be too long.

Most polyclinic practitioners had attended sessions during which the diabetes protocol was presented, and felt this method of introducing the guidelines was useful but criticized a lack of copies for non-physicians and for physicians arriving after the initial distribution. This protocol was not promoted to private practitioners.

Non-physician private practitioners, like some of their public colleagues, follow their own professional guidelines and were generally unaware of the more general ones issued by CCMRC and the Ministry of Health. A reason given was that the guidelines were not circulated to them. The idea of general guidelines for all healthcare providers was rejected because of the concern that physicians would use the small amount of information concerning the non-physician practice in the guidelines and usurp the role of non-physicians; a lack of involvement in their development; and because guidelines are quickly outdated, use valuable resources and are hard to remember. Patient brochures put out by drug companies were widely used and appreciated by pharmacists.

Practitioners strongly recommended that any new guidelines be heavily and repeatedly promoted (possibly by individual detailing), be kept short by using algorithms, updated regularly with loose-leaf additions, and have CD-ROM and online versions. Polyclinic practitioners recommended that there be a sign-in sheet at sessions promoting guidelines and attendance be mandatory.

A patient oriented version of the guidelines was welcomed by all. Patient materials should address local dietary habits and footwear. Versions aimed at healthy children and young adults stressing primary prevention were also recommended. Enthusiasm for patient oriented materials came despite complaints that patients do not read brochures or watch videos being presented in the waiting room. Patients were said to be only interested in material that was colourful, in large print, consisted mainly of pictures and showed people, foods and clothes/footwear that come from Barbados. Participants felt that materials should stress the importance of self-management, as well as complications of the diseases but make clear that life with disease could still be happy and healthy if you followed advice.

Practitioners also thought that a copy of the medical record could be given to patients, allowing them to see if they were achieving treatment goals, and to know what tests were due so that they budget accordingly if paying for tests in the private sector. This record could be taken to any practitioner visited allowing all practitioners to be fully informed of the patient's status. This "diabetes and hypertension passport" could be part of a patient version of the guidelines.

#### Special programmes for patients

Of the 8 polyclinics 3 had diabetes clinics, but only one a hypertension clinic. Of the remaining clinics 5 and 2 respectively had diabetes and hypertension educational programmes with one of these having an exercise programme. Staff at clinics without specific diabetes and hypertension clinics, expressed a desire to have them, and felt that they would improve care, but thought that a lack of resources, primarily human, was the barrier to having them.

The team approach, with all members of the team respecting and understanding the role of all others, and all caregivers giving the same, or at least, non-conflicting information, was recommended by non-physician private practitioners. "It does not help to have tension in the team". It was felt that doctors did not communicate well with non-physicians. They were often unapproachable, did not understand the role of other professionals and often did not refer to non-physicians even when their services were needed, and often tried to usurp their role. Non-physician practitioners felt that they spent more time with patients than doctors do, so may develop more rapport. Patients were more likely to divulge non-adherence to them, and this allowed the practitioner to better deal with misinformation and confront denial and fear. One polyclinic practitioner warned of patient confusion caused by the uncoordinated team approach. "The dietician may say to them that they need to eat more of a particular thing but the doctor or podiatrist may give different advice". Another said, "I believe that only the dietitian should be giving nutritional advice because that is my role and what I'm an expert in".

### Barriers

All polyclinic practitioners felt that they provided good quality care. Many of the reasons given for outcomes being less than ideal by polyclinic practitioners were related to patient factors despite the best efforts of practitioners.

#### Patient factors

*1. Patient denial *which was greater for diabetes than for hypertension, because of the greater associated stigma. Denial and stigma can cause patients to hide their disease. By not allowing others to see them taking pills, using special diets etc. they were less likely to do these things despite the educational efforts of practitioners. "You have diabetics in a home and the other relatives don't even know that the individual is a diabetic". One practitioner concluded "I think it's a denial thing, taking medication means I am sick, what does it solve when there ain't nothing wrong with me, I really don't have any diabetes".

2. A *lack of understanding *by patients about the disease or treatment modalities. This especially included knowledge on how to cook the right foods, the sources of salt in the diet, the medication regimen, the belief that medication is only needed when symptomatic, and the belief that medication caused side effects which in fact had other causes such as the disease itself and aging.

3. Patients being *unable to afford *the prescribed treatment and monitoring e.g. diet (particularly fruit and vegetables), exercise facilities or equipment, laboratory tests in the private sector and home BP and self blood glucose monitoring (SBGM) equipment.

4. Patients' *forgetfulness *and confusion primarily concerning medication regimens. Confusion might occur when changes were made to the regimen, the patient attended different doctors and had different regimens prescribed, and when the pharmacist made changes because of being low or out-of-stock of a prescribed medication.

*5. Side effects *such as impotence, frequency of micturition and fatigue caused by medications, particularly those used to treat hypertension. Patients in Barbados felt they must keep the practitioner happy and falsely report taking medication without problems rather than report side effects and requesting a change in regimen.

6. Patients' *religious beliefs or belief in alternative medicine*, which lead them to believe that they will be cured without treatment or through alternative treatment. Such beliefs were often not reported to practitioners.

7. Patients being "incapable of reversing 40 years of bad habits".

8. Patients' *lack of time *for cooking proper foods and the environment not being conducive to exercising.

9. Some patients' *fear of sticking themselves *prevented them from doing SBGM and/or from using insulin.

*10. Late presentation by patients *due to ignorance or denial, or non-adherence with advice following earlier diagnoses

It was stressed that non-adherence was more prevalent for hypertension than diabetes because patients were more likely to be asymptomatic, the sequellae of the disease seem to worry patients less, and the medications have more side effects. "A patient's blood pressure may be sky high and looking to have a stroke any minute and they simply continue on their merry way without a clue. So if you don't feel ill why would you go to the doctor? And that is the biggest barrier we face". "Some of them feel that hypertension carries a symptom and that they can feel it in their head, and some feel that their blood pressure is too low and that it slows them down". However, the stigma of hypertension is less than for diabetes so patients could accept the diagnosis with less difficulty.

Private sector physicians placed less blame for poor outcomes on lack of patient adherence, and more emphasis on the need to communicate with patients so that they could accept and understand their disease. "This is my job to make sure they are motivated-to do this I must understand the patient". Emphasis was placed on financial barriers in accessing care from podiatrists, dieticians and ophthalmologists, laboratory testing, "healthy" foods, exercise facilities and drugs not provided free e.g. those for hyperlipidemia. They particularly blamed long waiting times for appointments with public sector consultants and lack of feedback after the patient had been seen as a barrier to care for those who could not afford additional private care beyond the family physician. Results of diagnostic tests from the public system often took too long to be available. Free medication was not valued, so patients did not take them properly. Some felt that a very small charge would result in patients valuing them more. However, it was also felt patients preferred to take free and easily available medication rather than try to change lifestyle.

#### System barriers

*1. Lack of access to needed investigation tools and teaching aids *in the polyclinic setting such as haemoglobin A1c reagents, blood tubes required to carry out various tests, large and children's sized blood pressure cuffs, equipment for the podiatrists, and educational videos and models used by dieticians in teaching. In the private sector the cost of tests and care provided by dieticians, podiatrists and others was a problem.

*2. Lack of a reliable supply of medications*. Even though medication was free, and one month's medication should be dispensed at a time, patients were often given less than this at polyclinic pharmacies because of supplies running low. This required the patient to return for more medication before the end of the month, confusing the patient and contributing to non-adherence. It was reported that medications required to control the most resistant cases were not covered by the formulary and most patients could not afford them. The process to obtain them free was too burdensome. On the other hand, completely free medications could lead to patients undervaluing them.

*3. Lack of human resources *to deal adequately with the volume of patients presenting to polyclinics. This leads to inadequate interaction with and education of patients, unacceptable waiting times and an inability to visit patients in the community.

#### Barriers faced by patients

When asked about barriers that patients face when trying to maintain their health, practitioners listed the following:

*1. Difficulty obtaining time off work or from daily responsibilities *to attend clinic. This, combined with long wait times for both polyclinic visits and pharmacy supplies, means that patients often do not have the time to take in the education offered and may leave without medication, or may not return when medication should be renewed, leading to non-adherence.

*2. Cultural barriers *such as the typical Barbadian footwear; a diet that involves large meals (which are interpreted as a sign of love), and is high in salt and fat; the stigma of chronic disease (leading to patients hiding their disease and eating all foods to pretend they do not have disease); cultural attitudes to medication ("you only take it when you feel sick", "bush tea is good medication"); cultural attitudes to exercise (only for the young and must be a long hard workout or it is not exercise); cultural attitudes to obesity (men like fat women, if you are thin you have AIDS); high alcohol consumption by men; societal acceptance of the medical model where patients do not take responsibility for maintaining their health. Overeating at certain times such as Christmas and of seasonal fruits e.g. mangoes was seen as a problem for diabetes care.

3. A *lack of support *e.g. living alone without the means or energy to cook properly for oneself, or relying on an unsympathetic family cook who is unwilling to change the standard fare or cook a separate meal for the patient.

4. Patients were well aware of the *side effects of medication *and anticipated them. Medication package inserts, which overemphasized side effects, contributed to this problem.

### How the health care system could help providers improve the health of those with diabetes and hypertension

Suggestions included educating both the public and persons with the condition, screening programmes, providing free home monitors, and staffing issues.

1. Education campaigns should be similar to that for AIDS. Education on diet, exercise and obesity should be done in schools, and through the media (television especially). Messages should aim to get fruits and vegetables into the diet. Messages could be incorporated into popular songs, school skits, cartoons and other sites such as billboards and posters that appeal to young people. Group education/support programmes for new patients should be developed. Content should include patient responsibility for self-management including monitoring, and tackle the denial, stigma and avoidance of disclosure issues.

2. Screening programmes in malls, worksites and other public places can reduce the incidence of late diagnosis.

3. Home monitors should be available free to those who have been taught how to use them, to encourage responsibility and allow immediate feedback of the effects of diet and exercise.

4. The team approach should be encouraged by setting up case conferences involving all caregivers for problem cases, redesigning clinic space so team members can work together, bringing in resources to augment the team such as behaviour change experts and exercise experts.

5. Human resources should be increased and also better utilised so that more time could be spent with patients. By freeing nurses from tasks such as phlebotomy they would be able to do the tasks they were trained for (e.g. community outreach and home visits).

6. Physicians should be required to participate in continuing medical education (CME). There should be improved training for nurses in chronic disease management, with those involved in patient care and not managers getting priority to attend courses.

In addition to many of the recommendations above, private physicians thought that improving communication between the public and private sectors, facilitating timely care in the public sector for services patients could not afford privately, and providing financial support for patients who could not afford required tests, monitors and drugs would be helpful. Rapid access of private patients to the public system for diagnostic tests, dieticians, podiatrists, internists, ophthalmologists and cardiologists should be allowed. When patient care is shared there should be improved communication by the polyclinics and public sector specialists. Patients should not be scolded for crossing between the systems because, if they are, they will hide visits to one from the other, leading to poor care. The patient passport, or a centralized information system with data on each patient accessed by all caregivers would solve many problems. The latter was highly recommended if resources are available. There should be improved access to specially authorised drugs (drugs that are not automatically available free). The passport would help empower the patient to be the coordinator of his or her own care. One participant suggested more educational emphasis on the simpler, cheaper drugs for hypertension to counter the promotion of the expensive ones. The money saved could be used to fund the other recommendations.

### How wider society could help providers improve the health of those with diabetes and hypertension

Suggestions involved educational outreach to promote family support in managing the condition (cooking, encouraging exercise, giving insulin); a greater role for volunteer groups and retired persons in providing education, support, exercise groups and screening programmes; starting associations for hypertension and hyperlipidemia similar to the diabetes association or perhaps one association for all three conditions; the provision by the government of sidewalks and cycling lanes for safe exercise; healthy food choices at schools and work places; a tax on unhealthy fast food and an attempt to bring down the cost of healthy food by the government; a requirement that fast food outlets provide healthy alternatives; labelling of all food to include fat, salt and calorie content; encouraging a kitchen garden programme; time off by employers to attend appointments; and prominent persons with the disease should speak out to reduce stigma, and give hope that a good life can be had while living with chronic disease. In addition false and/or unhealthy messages given by the media on alcohol, cigarettes, candy, fast foods, herbal medicines, weight loss and the use of automobiles should be countered. It was suggested that the proceeds of a "fat tax" on unhealthy food could go to health care and to reduce the price of healthy foods.

## Discussion

This study showed that many practitioners did not use regional guidelines. It also showed that the family physicians, nurses, dieticians, podiatrists and pharmacists who comprise the health care team responsible for most primary diabetes and hypertension care have much to contribute both to guideline development and implementation.

Practitioners can become aware of guidelines by diffusion (distribution of information with unaided adoption) as in the case of CCMRC guidelines, by dissemination (communication of information to improve knowledge and skills) as was the case of the diabetes protocol for polyclinic practitioners, or by implementation (active dissemination including strategies to overcome barriers) [20]. In many cases the lack of use of guidelines would have been due to a lack of awareness that they exist, or to a lack of familiarity with the contents. Other reasons included a lack of agreement with the guidelines (may not give good care by following the guidelines), guideline related barriers (too long, and hard to remember), and patient, physician, and system barriers.

Practitioners thought that they provided a good quality of care. Actual practice as revealed by a chart audit conducted at the same time as this study revealed significant deficiencies in the frequency with which recommended processes of care were performed, and with the attainment of control targets [9,10]. Physicians often overestimate the quality of care they provide [25-27]. Without the benefit of an audit with feedback, practitioners may not realise how far short they fall from meeting targets set by the guidelines, and have no direction in how to improve their care. The creation of a patient version of the guidelines may also assist as patients will be in a better position to monitor their care and request interventions.

Physicians may attribute a less than ideal outcome primarily to patient non-adherence [27]. It has however been suggested that a major obstacle to better BP control is the failure of the physician to intensify therapy [28,29]. Higher levels of communication, guidance and familiarity with behaviour change techniques by physicians may be necessary to overcome patient factors such as denial; poor understanding of the condition, and treatment; the limitations of alternative medicine and religious beliefs; ingrained habits and fears; and lack of time management skills necessary for appropriate exercise and food preparation. There might possibly be a lack of self-efficacy (the belief that one can adequately perform the required process of care) or outcome expectancy (the process will produce the desired outcome) with regards to interventions aimed at these patient factors.

Understanding behaviour change models such as the transtheoretical model of behaviour change may help practitioners to focus less on patient failure, and more on the importance of matching interventions and expectations to the patient's readiness to change [30]. The understanding that sustained behaviour change is not usually a discrete single event but a gradual shift requiring varying amounts of time might lower physician frustration during the change process, and increase self-efficacy. Private sector physicians placed less emphasis on patient adherence, and a greater emphasis on a need to communicate with patients than polyclinic practitioners. It is possible that differences in patient characteristics and system factors including time and incentives might account for some of this difference between practitioners.

Practitioners identified system barriers, which centred on the availability, timely access and cost of the necessary resources for achieving good care. Implementation of guidelines would involve putting measures in place to correct these barriers. Some deficiencies might be easier to correct than others. Supplying an adequate number of appropriate size blood pressure cuffs should be simple, but to improve care, staff would have to use them. Free laboratory tests for private patients would be more costly, but even a modest decrease in the cost to patients might increase the frequency with which certain tests are done. Cost effective prescribing could save money. It is likely that physicians are exposed far more frequently to detailing of the more expensive drugs by pharmaceutical representatives, than to strictly academic presentations stressing evidence based use of lower cost drugs. Some of the barriers faced by patients would be beyond the scope of the individual practitioner to influence. However some issues such as difficulty taking time off work could be minimised by having specific appointment times in polyclinics and a better customer focus at pharmacies. Systems could be put in place to monitor waiting times and maximum waiting time targets set.

The interdisciplinary team approach with special clinics and programmes appeared to be valued by polyclinic practitioners. On the other hand private non-physicians did not think that their skills were properly utilised by physicians, and some feared that if physicians were more knowledgeable they would use their services even less. The integrated team approach as a model for the treatment of chronic illness is not a new concept. It may be more effective in improving outcomes than traditional face-to-face physician visits, but adoption requires a shift in how providers view their roles and relationships, both with patients and with professionals in other disciplines [31,32].

## Conclusions

Barbados must take a societal approach if it is to change sufficiently to improve the health of citizens living with diabetes and/or hypertension. The health care system alone cannot be expected to change lives and behaviours. However, the health care system must also change to improve the care of those with these diseases. This change can start with an intensive, multi-pronged implementation of new guidelines, including compulsory educational sessions for health care providers, academic detailing and audit and feedback techniques. But it must go beyond such efforts to include improved public and patient education, system improvements in the health care system and improved patient access to essential care.

## Competing interests

The authors declare that they have no competing interests.

## Authors' contributions

OPA, AOC participated in the conception and design of the study; the acquisition, and interpretation of data; and drafting and revising the manuscript critically. Both authors have read and approved the final manuscript.

## Pre-publication history

The pre-publication history for this paper can be accessed here:

http://www.biomedcentral.com/1471-2296/11/96/prepub
